# IL-1 and senescence: Friends and foe of EGFR neutralization and immunotherapy

**DOI:** 10.3389/fcell.2022.1083743

**Published:** 2023-01-12

**Authors:** Donatella Romaniello, Valerio Gelfo, Federica Pagano, Michela Sgarzi, Alessandra Morselli, Cinzia Girone, Daria Maria Filippini, Gabriele D’Uva, Mattia Lauriola

**Affiliations:** ^1^ Department of Experimental, Diagnostic and Specialty Medicine (DIMES), University of Bologna, Bologna, Italy; ^2^ Centre for Applied Biomedical Research (CRBA), Bologna University Hospital Authority St. Orsola -Malpighi Polyclinic, Bologna, Italy; ^3^ Division of Medical Oncology, IRCCS Azienda Ospedaliero-Universitaria di Bologna, Bologna, Italy; ^4^ National Laboratory of Molecular Biology and Stem Cell Engineering, National Institute of Biostructures and Biosystems (INBB), Bologna, Italy

**Keywords:** senescence, IL-1, EGFR, immunotherapy, moab, senotherapeutics, PD1 (programmed cell death protein 1)

## Abstract

Historically, senescence has been considered a safe program in response to multiple stresses in which cells undergo irreversible growth arrest. This process is characterized by morphological and metabolic changes, heterochromatin formation, and secretion of inflammatory components, known as senescence-associated secretory phenotype (SASP). However, recent reports demonstrated that anti-cancer therapy itself can stimulate a senescence response in tumor cells, the so-called therapy-induced senescence (TIS), which may represent a temporary bypass pathway that promotes drug resistance. In this context, several studies have shown that EGFR blockage, by TKIs or moAbs, promotes TIS by increasing IL-1 cytokine production, thus pushing cells into a “pseudo-senescent” state. Today, senotherapeutic agents are emerging as a potential strategy in cancer treatment thanks to their dual role in annihilating senescent cells and simultaneously preventing their awakening into a resistant and aggressive form. Here, we summarize classic and recent findings about the cellular processes driving senescence and SASP, and we provide a state-of-the-art of the anti-cancer strategies available so far that exploits the activation and/or blockade of senescence-based mechanisms.

## Introduction

The field of cellular senescence (CS) dates back to 1961, with the work of Hayflick and Moorhead, who proved that fetal fibroblasts derived from human tissue, displayed a limited replicative capability *in vitro* (Hayflick limit) (Hayflick and Moorhead, 1961). This discovery changed the dominant view that cultured cells would grow indefinitely, and for the first time, the proliferative difference between normal cells (or cell strain) and tumor cells (or transformed cells) was highlighted. Finally, in 1990 Calvin Harley demonstrated that telomeres shortening during aging was responsible for cellular senescence, a process that could be blocked in immortalized germ cells by maintaining a significant telomerase activity (Harley et al., 1990). Nevertheless, the telomere hypothesis alone was non-sufficient to explain the stochastic variation in cell division among the same cell population and in clonally derived cells. Nowadays, we count multiple mechanisms involved in cellular aging namely oxidative stress, DNA damage, radiations, and expression of some oncogenes among others (Gorgoulis et al., 2019). In this context, a plethora of DNA-related damages have been identified to culminate in the senescence phenotype.

**FIGURE 1:**
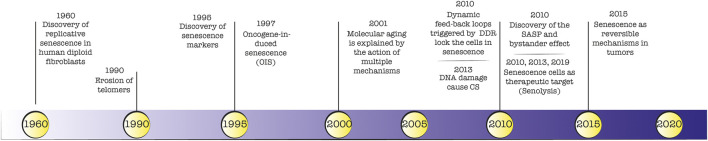
Timeline of senescence landmark discoveries.

A detailed timeline of landmark discoveries about senescence is depicted in Figure 1. The identification of markers for senescent cells *in vitro* and *in vivo* has been widely debated and to some extent is still ongoing. The first biological marker of senescence was identified in 1995 by Dimri and colleagues (Dimri et al., 1995), who reported a prominent β -galactosidase lysosomal enzymatic activity in senescent cells. During the last 2 decades, histological and molecular studies described several cellular senescence hallmarks. For example, the increased production of cell cycle arrest markers p53/p21 (Dulić et al., 2000; Beauséjour et al., 2003) and p16-pRB (Ben-Porath and Weinberg, 2005) axis, identified in 2000 and 2003 respectively, are considered defining traits of cellular senescence. Indeed, the cell cycle arrest observed in senescent cells represents a logical consequence of the lack of proliferation and DNA synthesis. For this reason, when absent KI67 and BrdU markers were considered signs of senescence. Also, markers related to cell size (enlarged cells) and morphology (flattened cells), DNA damage (yH2AX), ROS production, telomere shortening, and secretion of signal molecules became relevant for the identification of the senescence phenotype both *in vitro* and *in vivo*. The major limitation is the lack of a single, universal biomarker hampering the ability to detect senescent cells confidently. This reflects the heterogeneous phenotype that may differ from the triggering insult and the analysed cell type. Instead, a multi-marker approach can discriminate between stably arrested senescent cells and proliferating cells in order to evaluate the efficacy of senolytic agents (Gorgoulis et al., 2019). A comprehensive list of senescent markers (i.e., SA-β-galactosidase, p16^INK4a^, p21, morphology, cell size, SAHF formation) was reviewed by Gonzales-Gualda et al. (González-Gualda et al., 2021).

Most senescent cells undergo alteration activating the so called “senescence-associated secretory phenotype” (SASP), that involves the production of a large set of active molecules such as growth factors, proteases, cytokines, chemokines, and extracellular matrix components. The nature and composition of SASP are not unique and depend on the senescence trigger, cell type, environmental context and time elapsed since senescence initiation.

Moreover, the production of such molecules strongly impacts neighboring cells (bystander effect), amplifying and spreading the original generation of senescent cells. The effects of SASP on tissue homeostasis are dependent on multiple factors. SASP can exert a beneficial or even essential effect on embryonic patterning, tissue repair, wound healing, cell stemness and plasticity, hepatic fibrosis control, immune surveillance, and containment of uncontrolled cell growth. On the other hand, SASP can have highly detrimental outcomes when affects non-damaged, healthy cells, with negative repercussions in terms of chronic inflammation (Kowald et al., 2020), degenerative diseases, and carcinogenesis. Broadening the molecular characterization related to senescence and the link with age-related diseases would allow intercepting senescent cells as a therapeutic target. For this reason, over the last 5 years, pharmacological agents have been employed to eliminate (senolityc) or ameliorate the detrimental cell-nonautonomous effect (senomorphic) of senescent cells with promising results (Di Micco et al., 2021).

## Senescence phenotype in development and healthy tissue

The beneficial role of senescence is mainly attributable to tumor suppression activity. Nevertheless, this positive role clashes with the discovery of SASP and its bystander effect in neighboring cells, which promotes chronic inflammation and tumor progression. Indeed, sustained IL-1 secretion may be also responsible for the tumor senescence escape, reflecting into the attenuation of the senescence surveillance, thus leading to tumor formation. A mechanism already reported as SASP mediated paracrine senescence, which can impact on tumour suppression and senescence *in vivo* (Acosta et al., 2013)*.*

Embryonic development, tissue repair (Reyes de Mochel et al., 2020), and remodeling displayed a form of senescence characterized by the activation of molecular pathways not related to those triggered by DNA damage response. In these contexts, senescence displayed specific lineage-dependent molecular mechanisms that vary based on the tissue/organs involved (Flavell et al., 2010; Muñoz-Espín et al., 2013).

A gene expression analysis showed that during development senescence is strictly dependent on the expression of p21 and regulated by TGFβ/SMAD and FOXO/PI3K signaling pathways, particularly in the epithelia of the mesonephros and endolymphatic sac (Muñoz-Espín et al., 2013; Gibaja et al., 2019). Also, developmental senescent p21-expressing cells can re-enter the cell cycle and contribute to the adult tissue (Li et al., 2018).

In differentiated cells, senescence occurs in a programmed manner with a physiological role. For example, megakaryocyte and placental syncytiotrophoblast undergo senescence as part of their physiological maturation programs. In megakaryocytes, thrombopoietin recapitulates the oncogene-induced senescence by inducing a sustained activation of the RAS/MAPK axis. One hypothesis is that senescence, in this context, could have a key role in the post-mitotic arrest, thus inhibiting proliferation, without inducing cell death (Besancenot et al., 2010).

## Mechanisms of senescence in EGFR inhibition

EGFR is a powerful oncogene commonly found overexpressed or catalytically altered by gene mutations in many types of solid tumors (Brennan et al., 2013; Maron et al., 2019; Srivastava et al., 2019; Marrocco et al., 2021). EGFR (also known as ERBB1/HER1) belongs, together with HER2 (ERBB2), HER3 (ERBB3), and HER4 (ERBB4), to the tyrosine kinase family of ERBB receptor (Lemmon and Schlessinger, 2010). Ubiquitously expressed, it plays a crucial role in embryogenesis and post-natal development (Chen et al., 2016). The binding with one of its ligands EGF, Transforming Growth Factor (TGF)-α, Heparin-binding Epidermal Growth Factor (HBEGF), Betacellulin, Amphiregulin, Epiregulin, Epigen, and the recently described Connective Tissue Growth Factor determines a conformational change of the receptor from an inactivated monomeric form into an asymmetric dimeric one forming homo or heterodimers with the other members of the family. The receptor activation results from trans-autophosphorylation, performed by the “receiver” kinase, of certain tyrosine residues present in the C-terminal tail of the “activator” kinase (Kovacs et al., 2015). This process allows the recruitment of specific adaptors and propagates EGFR signaling towards several downstream pathways, which are essential regulators of cell proliferation, migration, differentiation, survival, and metabolism: the Ras/Raf/Mitogen-activated protein kinases (MAPK) pathway, the phosphatidylinositol 3-kinase (PI3K)/AKT8 virus oncogene cellular homolog the (AKT)/Mammalian target of rapamycin (mTOR) pathway and the signal transducer and activator of the transcription (STAT) are the most important ones (Yarden and Sliwkowski, 2001; Jorissen et al., 2003; Wee and Wang, 2017).

There are two main therapeutic approaches to target EGFR: humanized monoclonal antibodies (moAbs) or tyrosine kinase inhibitors (TKIs). The first class, binding to the extracellular domain, is able to recruit and activate immune-effector cells, to neutralize signal activation by blocking ligand binding and inducing receptor internalization and degradation (Marrocco et al., 2019). They usually have a long half-life requiring only one administration per week. The first moAb anti-EGFR approved was cetuximab (Erbitux™) used in clinical practice in combination with chemotherapy for RAS wild-type advanced colorectal cancer (CRC) and with radiotherapy or chemotherapy for locally advanced patients unfit for platinum, and for recurrent/metastatic patients, without PD-L1 expression, with head and neck squamous cell carcinoma (HNSCC), respectively (Baselga et al., 2000). Since the discovery of activating EGFR mutation, TKIs are currently the standard of care for advanced NSCLC patients which provide strong anti-tumor activity compared to platinum-based chemotherapy (Hanna et al., 2017; Yoneda et al., 2019). TKIs are small molecules, mutant selective, that compete with adenosine triphosphate (ATP) binding of the intracellular kinase pocket preventing signal transduction. Orally available, they require daily administration due to their short half-life. Erlotinib and gefitinib are the first generations of reversible TKIs approved, however, the onset of acquired resistance led to the design of new generations of irreversible-TKIs, which covalently bind the receptor, like afatinib (second generation) and osimertinib (3^rd^ generation). The latter showed to be superior even in first-line treatment, with less toxicity compared to afatinib, which targets also the wild-type EGFR (Park et al., 2016; Soria et al., 2018). The inevitable drug resistance represents a major challenge for successful treatment and many efforts have been done for a deeper understanding of the tangled gear of recurrence. In the oncologic field, it has been shown that even tumor cells can undergo senescence in response to stressful stimuli derived from anticancer treatment exposure, known as therapy-induced senescence (TIS). Indeed, genotoxic agents, like etoposide or cisplatin, were shown in several cell types to induce TIS at lower doses (Ewald et al., 2010).

**FIGURE 2:**
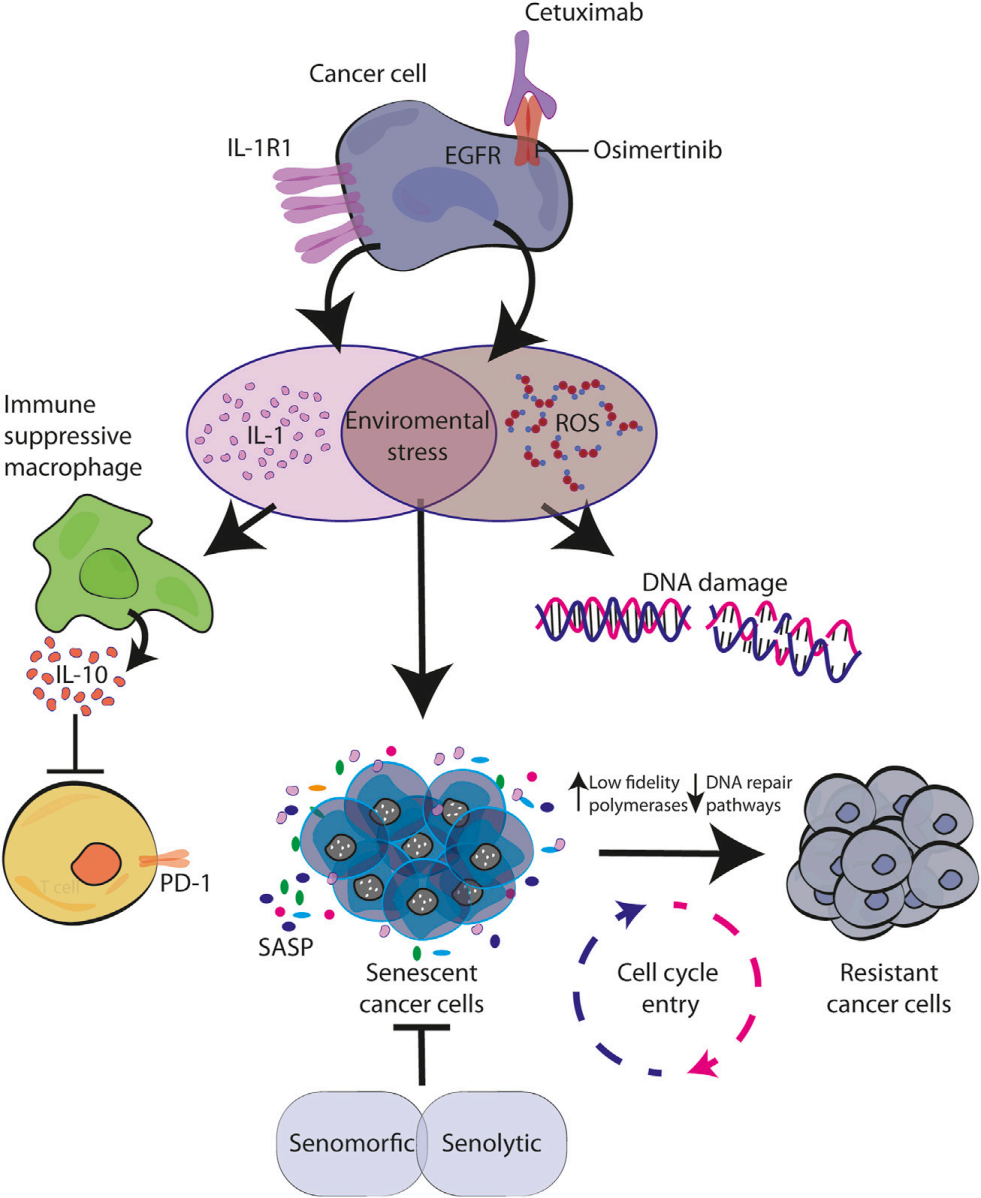
Senescence activated by environmental stress causes resistance to EGFR blockade. EGFR targeted therapy activates stress response in cancer cells characterized by IL-1 and ROS production. By creating a pro-inflammatory environment with immunosuppression function and by inducing DNA damage, they both contribute to the establishment of senescence in autocrine and paracrine manner. During this time, the increased levels of error-prone DNA polymerases allow cell cycle re-entry of drug-resistant mutagenic variants which ultimately converge for tumor relapse (Kaplanov et al., 2019; Singh et al., 2021; Romaniello et al., 2022).

However, recent findings demonstrated that also targeted therapies including anti-EGFR inhibitors are able to trigger TIS (see next paragraphs). This effect is caused either by TKIs or moAbs (Hotta et al., 2007; McDermott et al., 2019). In our work we explored the effect of cetuximab in colorectal cancer *in vitro* models and the antibody was able to increase senescence markers. In addition, our investigation also revealed that cetuximab was acting by inducing the production of the pro-inflammatory cytokine IL-1 and released in the microenvironment (Romaniello et al., 2022). Considering the known role of IL-1 in inducing senescence, is tempting to consider it as one of the mechanisms of action for CTX, that will achieve a cell cycle arrest by immediate autocrine production of IL-1 (Figure 2). Further analysis and experiments are needed in order to understand all the dynamic puzzles, which might be different between TKIs and moAbs, that in the end convert senescence cells into resistant ones.

## The two sides of a coin: IL-1 and senescence-induced response and resistance to EGFR blockage

In cancer therapy, systemic treatments with chemotherapeutic agents engage a sustained cell cycle arrest. These stresses proceed by inducing a double-strand break (DSB) and persistent DNA damage induces several cellular dysfunctions such as an imbalance of signaling, oxidative stress, proteotoxicity, and altered chromatin structure resulting in cellular senescence (Mongiardi et al., 2021).

In 1970, it was reported that 5-fluorouracil causes a senescence phenotype in fibroblast (Holliday and Tarrant, 1972) and during the last 5 decades a plethora of anti-cancer agents able to induce senescence have been identified. Several drugs causing DNA damage have been reported as inducers of senescence, among them chemotherapeutic agents like topoisomerase inhibitors, alkylating agents, platinum-based agents, antimetabolites microtubule inhibitors and hormonal therapy (Saleh et al., 2020). More recently targeted therapies, such as tyrosine kinase inhibitors and monoclonal antibodies were described to cause DNA damage (Russo et al., 2019; Saleh et al., 2020) and senescence. For example, lapatinib, an EGFR-HER2 inhibitor, when employed in HER2-positive drug-sensitive breast cancers was able to induce senescence (McDermott et al., 2019). Also, the pan-HER neratinib and afatinib proved to induce senescence, corroborating a role of the ErbB receptors family in TKIs-induced senescence (McDermott et al., 2019; Chaudhary et al., 2021). Previous observations from our laboratory shown that CRC cells exposed to monoclonal antibody anti-EGFR, displayed an increased expression of EMT markers and an array of cytokines, IL-1α, IL-1β and IL-8 (Gelfo et al., 2016). Interestingly, the CTX-treated CRC cell line displayed a senescence phenotype, and conversely, IL-1 neutralization was able to revert the CTX-resistant cells into a senescence-mediated growth arrest (Romaniello et al., 2022).

Stemming from these observations, we drew a model for IL-1 production by CTX, which drives cancer cells into a cell cycle arrest, reminiscent of a pseudo-senescence state, which indicates a reversible senescence, employed by the cancer cells to protect themselves from drug toxicity, to finally re-enter into a proliferative state (rebound grow) through downregulation of p53 or p16 INK4A, expressing surviving, stemness marker or restoring nuclear and morphological structure (Chakradeo et al., 2015; Mastri et al., 2018; Saleh et al., 2018). In this context, chronic exposure to IL-1 would reprogram CTX-resistant cells in a poorly differentiated phenotype, compatible with EMT associated to cell cycle re-entry, tumor relapse, and aggressiveness. Also, Gefitinib and Erlotinib, two anti-EGFR TKIs, have been correlated with the induction of senescence in EGFR-mutant and non-mutant NSCLC cell lines (Hotta et al., 2007; Wang et al., 2011; Sugita et al., 2015). In details, higher concentrations of Gefitinib were shown to induce extended apoptosis in EGFR-mutated PC-9 cells and EGFR wild-type cells, whereas clinically relevant concentrations of gefitinib induced prominent, premature senescence, characterized by an increase of p16^INK4A^, p21^WAF1/Cip1^, and p27^Kip1^ levels and subsequent G_1_ cell cycle arrest (Hotta et al., 2007). Conversely, these effects were not observed in gefitinib-resistant cells. Among monoclonal antibodies, pertuzumab and trastuzumab were also shown to promote senescence in breast cancer models, particularly when combined with pro-inflammatory cytokines like TNF-α and IFN-γ (Rosemblit et al., 2018). Interestingly, targeted therapies to ErbB proteins present transcriptional modifications and microenvironment changes, that may contribute to therapeutic failure. In this context, TIS could play a key role in the mechanism of action of ErbB targeting agents and may be a reasonable route for the emergence of resistance, although this remains to be proven.

## The senescence-associated secretory phenotype (SASP): Role for IL-1

SASP has been described as a temporally regulated dynamic program that can be divided into an initial rapid phase followed by an early self-amplification phase, eventually leading to a late mature phase (Kumari and Jat, 2021). During the last decade, extensive analysis of SASP components identified a plethora of molecules secreted, such as pro-inflammatory cytokines, chemokines, growth, angiogenic factors, proteases, bioactive lipids, extracellular factor components and metalloproteases as components of SASP (Coppé et al., 2010; Hernandez-Segura et al., 2017). The SASP components depend on the inducer of senescence, duration, environment, and cell type. SASP factors can reinforce and spread senescence in a non-cell-autonomous fashion, including the manifestation of senescence in healthy, proliferating-competent cells and stimulating changes in the phenotype of neighboring cells (Coppé et al., 2008; 2010) a phenomenon referred to as paracrine senescence, that may reinforce senescence, activates immune surveillance and paradoxically also has pro-tumorigenic properties (Acosta et al., 2013). Among the component of SASP, the secretion of pro-inflammatory cytokines including IL-1α, IL-6, and IL-8 are controlled by NF-kB and CEBPβ in an autocrine feedforward manner (Huggins et al., 2013; Zhang et al., 2018). IL-1α secreted by endothelial cells, fibroblasts, and chemotherapy-induced senescent epithelial cells plays a key role in the establishment of senescence exerting also harmful effects on the tissue microenvironment. The tissue microenvironment might affect tumor development and growth. Indeed, tumors producing a pro-inflammatory environment can promote cancer progression. For example, an inflamed tumor microenvironment (TME) favors migration and expansion of immunosuppressive components namely myeloid-derived suppressor cells (MDSCs), tumor-associated macrophages (TAM), tumor-associated neutrophils (TAN), regulatory B (B_reg_) cells and Th_17_ (Coppé et al., 2010; Gelfo et al., 2020). Chronic inflammation and an infiltrated immunosuppressive TME promote cancer initiation, progression, and resistance to therapy. Interestingly, senescence surveillance of pre-malignant cells usually limits cancer development, by activating CD4^+^ T-cell, which are important for tumour suppression *in vivo*, representing an extrinsic component of the senescence anti-tumour barrier. The senescence inducer IL-1 is known to be a cytokine triggering T-helper immune response and being an innate mediator of T cell immunity. IL-1 may also enhance immune surveillance, which, in turn, accounts for the clearance of senescent cells. This was, for example, exploited to turn immunologically “cold” tumors into T cells infiltrating one, thus creating the conditions for boosting PD-1 checkpoint activity (Ruscetti et al., 2020; Hao et al., 2021). Sustained IL-1 secretion may be also responsible for the tumor senescence escape, reflecting into the attenuation of the senescence surveillance, thus leading to tumor formation. A mechanism already reported as SASP mediated paracrine senescence, which can impact on tumour suppression and senescence *in vivo* (Acosta et al., 2013)*.*

Senescent cells induced by irradiation, drug treatment, oncogenic stimuli, and other stressful conditions cause adverse effects on cancer cells and surrounding tissues (Vernot, 2020). As mentioned, the SASP of a particular senescent cell type may have a tumor-promoting effect depending on the host tissue. For instance, high levels of the pro-inflammatory cytokines IL-1, IL-6, and IL-8 are among the main soluble factors found in the SASP, responsible for increasing the invasiveness of a panel of breast cancer cell lines (Coppé et al., 2008); or for impairing the response to EGFR neutralization by monoclonal antibody (Gelfo et al., 2016; 2018; 2020) and kinase inhibitors (Stanam et al., 2016). However, results are controversial since IL-1α alone was also reported to increase and predict the efficacy of anti-EGFR in head and neck squamous cell carcinoma (Espinosa-Cotton et al., 2019). Of note, SASP factors were also found to alter epithelial differentiation and induce EMT (Coppé et al., 2008). Moreover, the SASP reinforcing senescence, activating immune surveillance has been paradoxically linked to pro-tumorigenic properties. In this context, the expression of the SASP is controlled by inflammasome-mediated IL-1 signalling. The inflammasome has been considered both a pro-carcinogenic and anti-carcinogenic factor, as it suppresses immunosurveillance and NK cell–and T cell–mediated antimetastatic actions; it favours tumor cell aggressiveness and neoangiogenesis, but also limit carcinogenesis by sustaining innate immune reactions against potentially carcinogenic microbiota; and facilitating antitumor immune responses (Zitvogel et al., 2012).

## Senescence upon DNA damage response (DDR)

Both senescence intrinsic factors, namely telomere shortening or oxidative damage as well as extrinsic factors, namely genotoxic drugs and ionizing radiation, can trigger senescence as a DDR (D’Adda Di Fagagna, 2008; Kumari and Jat, 2021). The presence of a single-stranded DNA or the generation of double-strand breaks (DSBs) activates specific protein kinases sensors (ATR, ataxia telangiectasia and Rad3-related or ATM, ataxia-telangiectasia mutated) causing the *cis* phosphorylation of H2AX histone (yH2AX) (Shiloh, 2006; Zou, 2007). Finally, the recruitment of several DDR mediators at the site of DNA damage culminates in the activation of decision-making factors, such as p53 and the cell-division cycle 25 (CDC25) phosphatases (Mailand et al., 2000; Turenne et al., 2001). All these events induce the formation of detectable nuclear DDR foci and a stable cell cycle arrest when the damage is irreparable. There is some evidence that demonstrated cells undergoing senescence even by simply colocalization of DDR sensors without DNA damage (Bonilla et al., 2008). In the case of replicative senescence, the telomere shortening is responsible for the loss of ATM-ATR inhibitors recognized as DNA breaks at the end of the chromosomes (D’Adda Di Fagagna et al., 2003). During oncogene-induced senescence (OIS), normal cells accumulate DNA damage due to their hyperproliferative activity and allow the recruitment of DDR machinery (Di Micco et al., 2006; 2007). Activations of RAS, BRAF, E2F1, and loss of PTEN are an example of oncogenes able to induce persistent cell division and DNA damage pushing cells into senescence (Di Micco et al., 2006). Thus, transient inactivation of DDR checkpoint genes allows cellular proliferation by blocking senescence, while their persistent activation may have a tumor suppressive effect (Visconti et al., 2016).

In tumor cells, it is known that chemotherapeutic drugs directly induce DNA damage. Indirectly, even targeted therapy against oncogenic signaling, such as EGFR and BRAF inhibitors, have demonstrated to trigger cellular stress and consequently DNA damage. For example, Russo et al. (2019) described in CRC models that treatments with cetuximab (moAb anti-EGFR) or dabrafenib (BRAF inhibitor) increase ROS production, down-regulate DNA-repair pathways and up-regulate low-fidelity polymerases. Similarly, this mechanism has been proved for EGFR-mutated NSCLC treated with TKIs (Noronha et al., 2022). These mutagenic mechanisms able to confer cell plasticity and drug tolerance induced by therapy-related stress may be correlated with the increase in IL-1 production under CTX treatment, coherent with a therapy-induced post-senescence previously described by our laboratory and others (Milanovic et al., 2017). A CTX-induced proinflammatory environment triggers cellular stress, consistent with ROS production and DNA damage, and the temporary establishment of senescence, a reversible state through which persistent cells are able to adapt and survive thanks to mutagenic plasticity (Figure 2).

## Senescence to survive: Reversible mechanism and plasticity

Cellular plasticity identifies the ability of cells to change phenotypic identity. Initially described as the main signature in embryonic cell differentiation, to date, it has been widely detected in adult cells both during physiological and pathological conditions.

The senescence has aroused particular interest in its involvement in cell plasticity in the TME. In general, senescence results in stable cell cycle arrest thus functioning as a potent defense mechanism against cancer (Hernandez-Segura et al., 2018). However, cancer cells can escape this tumor-suppressive state, highlighting senescence as a dynamic modification (Milanovic et al., 2017; Lee and Schmitt, 2019). Several investigators are currently trying to identify genes responsible for this plasticity, mostly through RNA interference-based or CRISPR (clustered regularly interspaced short palindromic repeats)–Cas9 (CRISPR-associated protein 9)-based senescence screens (Wang et al., 2017). For instance, it has been observed that the acute loss of all three members of the Rb family allows RAS-senescent fibroblasts to re-enter the cell cycle (Huang et al., 2003). Yu et al. (2018) demonstrated that two different types of active histone H3 lysine 9 (H3K9) demethylases, LSD1 and JMJD2C, override oncogenic RAS or B-RAF-induced senescence, by producing E2F target genes and enabling transformation. Conversely, blocking H3K9 demethylases restores senescence and controls tumor growth. Moreover, p53 was shown to be necessary for therapy-induced senescence in several malignant tumors (Milanovic et al., 2017). Nevertheless, human cancers have been shown to express EMT-TFs that are capable to repeal senescence effectors (p53, Rb, and p16^INKA4^) and interact with oncogenic signals to fully induce an EMT program and acquire invasive properties (Ansieau et al., 2008; Tran et al., 2012). In this regard, not only intrinsic senescence in cancer cells and its reversibility is crucial for disease progression, but any change in the tumor stroma could also affect tissue homeostasis and contribute to the aggressive outcome of cancer.

Recent evidence indicates that, at least in the context of tumor formation and anticancer therapies, the establishment of cellular senescence might involve epigenetic mechanisms reprogramming cancer cells towards a certain degree of stemness in a cell-autonomous fashion, with the potential to develop more aggressive tumors (Milanovic et al., 2017).

## Immunosenescence, IL-1 and immune checkpoint

Firstly introduced by Walford in 1994, immunosenescence, regarding both adaptive and innate immunity, is characterized by a readjustment of the microenvironment composition, including the complex cytokines landscape (Franceschi et al., 1999). In elderly people, bone marrow, thymus, and lymph nodes undergo morphological atrophy reducing their size and affecting the immune cells’ diversity. This process seems to be associated with decreased expression of sex hormones and an increased level of the hematopoietic growth factor IL-7, released by the stromal cells (Aiello et al., 2019). Therefore, the immune response against pathogens and tumor cells declines and the stromal cells network results disorganized (Becklund et al., 2016; Ruhland and Alspach, 2021). Also, macrophages and neutrophils reduce their phagocytosis activity and cytokine production. Even the quality and quantity of B and T cells are affected with a low number of peripheral naïve cells compared to young individuals due to the involution of the primary lymphoid organs. On the contrary, the number of relative late-differentiated memory T-cell subset increases over time, due to long-time exposure to pathogens and is found in replicative senescence (Chou and Effros, 2013). It has been demonstrated that those memory T-cells are characterized by CD28 negativity, co-stimulatory molecules, shorter telomeres, and by the expression of the receptor-programmed cell death protein PD-1. Thus, immunosenescence is strictly correlated with the development of aging diseases including tumorigenesis and the response to immune checkpoint inhibitors (ICIs) (Ferrara et al., 2017; Palmer et al., 2018; Thomas et al., 2020). Palmer et al., by analyzing 100 tumor distribution, confirmed the role of immunity in tumor growth (Palmer et al., 2018). As well, Pelissier Vatter et al. (2018) studying mammary epithelial cells from 57 different women, found a correlation between the high level of progenitor cells with increased cancer risk. However preclinical, and clinical data are still controversial about the role of senescence in tumor development. Indeed, different studies have shown that senescence impairs cancer growth and invasiveness improving cancer prognosis, in comparison with younger patients (Pili et al., 1994; Fisher et al., 1997), reflecting the conflicting outcomes detected in elderly patients treated with immunotherapy approaches. On the other hand, immunotherapy, by controlling the immune system through specific moAbs (ICIs) able to block inhibitory receptors or the expression of specific proteins, has marked a paradigm shift in the therapeutic strategy of cancers. PD-1 downregulating T-cell responses, and CTLA-4, a T-lymphocyte antigen able to suppress T_regs_ functions, are examples of ICIs modulated by specific moAbs for an anti-cancer effect. Thus, immunosenescence may influence the idea to activate an immune response against tumor cells in elderly patients. Clinical studies in metastatic melanoma using Nivolumab, a moAb anti-PD-1, and ipilimumab, moAb anti-CTLA-4, improved response independently on age. Controversial data concerning the immune response in elderly patients are reported in many tumors and clear evidence is still lacking (Elias et al., 2016).

In this context, IL-1β, has also been shown to play an important role in tumor-mediated inflammation. IL-1 is a pro-inflammatory and immunostimulatory cytokine, extensively present in the tumor microenvironment. Since widespread inflammation supports invasiveness as well as inhibits anti-tumor immune responses, IL-1 targeting has been thought to be a possible immunotherapeutic approach. Specifically, great interest has been given to IL-1β which is increased in several cancers and has proven to promote tumorigenesis, tumor invasiveness (Zhou et al., 2022) and immunosuppression (Krelin et al., 2007; Carmi et al., 2013; Voronov and Apte, 2020; Zhou et al., 2022).

Nevertheless, Novartis’s IL-1β-neutralizing antibody canakinumab alone failed in a phase III trial in previously untreated NSCLCs, despite the striking results showing that targeting the interleukin-1β pathway significantly reduces lung cancer incidence and mortality (Ridker et al., 2017).

By using a mouse breast cancer model with IL-1β deficiency or upon treatment with anti–IL-1β Abs, Kaplanov et al. showed that blocking IL-1β induces tumor regression, improves antitumor cell immunity and synergizes with anti-PD-1 incrementing its action (Kaplanov et al., 2019). So far, these data not only confirm its pivotal role in tumor progression, but also suggest a possible role for anti-IL-1β as a checkpoint inhibitor (Lee et al., 2022).

Finally, SASP was shown to have remodelling activity on the tumor microenviroment, raising the possibility to exploit it to improve susceptibility to otherwise ineffective chemo- and immunotherapies. For example, SASP induced therapy targeting endothelial cells was reported to stimulate the access of CD8^+^ T cells into otherwise immunologically “cold” tumors, thus creating the condition for boosting PD-1 checkpoint blockade activity (Ruscetti et al., 2020)**.** Similarly, in ovarian cancer, therapy-induced SASP was suggested to overcome resistance to immune checkpoint blockade. Indeed, by transferring cisplatin-induced SASP and boosting senescence, it was possible to sensitizes ovarian tumor to anti-PD-1 antibody and improving the survival of tumor-bearing mice in an immunocompetent, syngeneic model, probably thanks to the infiltration of activated CD8^+^ T cells and dendritic cells in the tumor surrounding (Hao et al., 2021). Sensitization of ovarian tumor to immune checkpoint blockade by boosting senescence-associated secretory phenotype). For an extensive overview, we suggest to refer to (Ruhland and Alspach, 2021).

## Senolytic agents as therapy

As discussed above, the appearance of therapy-induced senescence after treatment is now a widely known and established phenomenon. Considering its key and detrimental role in tumor progression, the discovery of drugs capable to eliminate therapy-induced senescent cells represents a new promising field for anticancer therapies. There are two main categories of senotherapies: senolytic drugs, which preferentially cause the death of senescence cells selectively, and senostatic (or senomorphic) drugs which inhibit senescence indirectly by suppressing the release of SASP factors. Although it is a new field of research and the knowledge of the exact mechanism of action at the molecular level is limited, both groups are promising with a particular interest for senolytic drugs, in combination with chemotherapy. Senescent cells are characterized by changes in chromatin structure, leading to alterations in gene expression, which in turn can affect fundamental cellular processes, like apoptosis. In details, senescent cells often exhibit elevated levels of the anti-apoptotic BCL-2 family proteins (Yosef et al., 2016), which has been intensively studied as a possible target in senolytic therapy.

With this purpose, several molecules have been studied, dasatinib a tyrosine-kinase inhibitor targeting several tyrosine kinases among which Bcr-Abl and the SRC kinase family, which has been shown to be effective in killing senescent cells. Moreover, Quercetin, a natural flavonoid, appears to have senolytic activity, acting on anti-apoptotic protein BCL-XL through inhibition of upstream pathways, including PI3K. A combination of both (D + Q) was shown to impair senescent cell levels in various *in vitro* and *in vivo* mouse models. In aged mice, the co-administration resulted in senescence cell removal, improved cardiovascular function and survival (Zhu et al., 2015; Paez-Ribes et al., 2019). Nonetheless, no clinical data are available so far on the effectiveness of dasatinib and quercetin co-treatment, although it showed promising results in pulmonary fibrosis, a life-threatening disease associated with the development of senescent cells in the lungs (Justice et al., 2019). However, the lack of mechanistic insights on how these drugs induce senolysis still represents an obstacle to their use in the clinical practice.

On the other hand, targeting SASP also represents a possible strategy to interfere with senescence. Indeed, senomorphics display no effect on senescent cell proliferation but are involved in the expression of factors that regulate specific biomarkers of senescence. For example, Apigenin and Kaempferol, two flavonoids structurally related to Quercetin, proved to act as senotherapeutic agent through SASP factors inhibition (Lim et al., 2015). Another attempt was made by testing approved drugs, like glucocorticoids, corticosterone and cortisol, which were found to be a potent suppressor of the senescence phenotype (Laberge et al., 2012).

As noted previously, combination therapies may also hold great potential, especially since they can prevent the emergence of resistance, by targeting on one side the oncological driver and on the other side the senescence bypassing pathway, responsible for a failed response. Table 1 reports a list of the ongoing clinical trials employing both senolytics and senomorphics agents, combined with anti-EGFR therapies.

**TABLE 1:** Listed are all clinical trials with senolytics and senomorphics combined with anti-EGFR therapies applied to different tumor types. Radiotherapy (RT); Chemotherapy (CT); Non-Small Cell Lung Cancer (NSCLC); Head and Neck Squamous Cell Carcinoma (HNSCC); Glioblastoma Multiforme (GBM); Triple Negative Breast Cancer (TNBC).

Drug combination with senolitycs	Target	Tumor type	ClinicalTrial.gov identifier (NCT)
Cetuximab + Dasatinib	EGFR + SRC Kinase	Advanced solid tumors	NCT00388427
Afatinib + Dasatinib	EGFR + SRC Kinase	NSCLC	NCT01999985
Cetuximab + Dasatinib + FOLFOX	EGFR + SRC Kinase + CT	Metastatic Colorectal Cancer	NCT00501410
Osimertinib + Dasatinib	EGFR + SRC Kinase	EGFR-mutated Advanced NSCLC	NCT02954523
Erlotinib Hydrochloride + Dasatinib	EGFR + SRC Kinase	Advanced	NCT00895128
		Tumors	
Cetuximab + Dasatinib + RT w/o Cisplatin	EGFR + SRC Kinase/CT	HNSCC	NCT00882583
Erlotinib Hydrochloride + Dasatinib + Gemcitabine Hydrochloride	EGFR + SRC Kinase + CT	Metastatic	NCT01660971
		Pancreatic	
		Cancer	
Cetuximab + Dasatinib	EGFR + SRC Kinase	Colorectal Cancer	NCT00835679
Erlotinib + Dasatinib	EGFR + SRC Kinase	Recurrent NSCLC	NCT00444015
Erlotinib + Dasatinib	EGFR + SRC Kinase	Glioblastoma and Gliosarcoma	NCT00609999
Cetuximab + Dasatinib	EGFR + SRC Kinase	HNSCC	NCT01488318
Erlotinib + Dasatinib	EGFR + SRC Kinase	Glioma	NCT02233049
Erlotinib + Navitoclax	EGFR + BCL-2	Solid Tumors	NCT01009073
Osimertinib + Navitoclax	EGFR + BCL-2	EGFR-mutant Advanced NSCLC	NCT02520778
Lapatinib + Vorinostat	HER2/EGFR + HDAC	Breast Cancer	NCT01118975
Erlotinib + Vorinostat	EGFR + HDAC	EGFR-mutant Advanced NSCLC	NCT00503971
Erlotinib + Vorinostat	EGFR + HDAC	EGFR-mutant Relapsed NSCLC	NCT00251589
Erlotinib + Vorinostat + Temozolomide	EGFR + HDAC + CT	Recurrent GBM	NCT01110876
Gefinitib + Vorinostat	EGFR + HDAC	EGFR-mutant NSCLC	NTC02151721

Drug combination with senomorphics	Target	Tumor Type	ClinicalTrial.gov Identifier (NTC)
Erlotinib + Metformin	EGFR + metabolism (mitochondrial complex I)	TNBC	NTC01650506
Erlotinib + Metformin + Gemcitabine	EGFR + metabolism (mitochondrial complex I) + CT	Pancreatic Cancer	NTC01210911

A one-two punch model where pro-senescence therapy was followed by a senolytic therapy was proposed by Bernards’ laboratory (Wang et al., 2017). Sequential drug treatment regimens would allow for a combination of a wider range of drugs, avoiding direct toxicity which is certainly one of the most important limitations of combinatorial strategies. However, the spectrum of drugs with high efficacy in the induction of senescence is still limited, and agents with clear selectivity for cancer cells compared to normal cells are not available so far. Considering the tumor heterogeneity, a further challenge to be solved is the lack of broadly acting senolytic drugs; indeed, the efficacy of senescence induction in tumors may be impaired by tumor heterogeneity, which represents the primary source of inconsistent pharmacological responses. Similarly, we should consider the effects of the SASP of senescent cells on the local tumor microenvironment as well as on the immune system. Overall, given the relevance of senescence-based therapies and the many questions raised, intense and well-orchestrated multi-level studies will certainly be needed to show their true potential.

## Discussion

It is well consolidating that under drug pressure, tumor heterogeneity and cell plasticity, play an important role in generating multiple escaping routes and establishing resistance to therapies.

We previously described that in a pro-inflammatory environment, IL-1 often triggers cellular stress activating senescence. In details, anti-cancer drugs targeting ERBB family proved to induce senescence by triggering IL-1 production and release. This may represent the friendly role for senescence in inducing cell cycle arrest. On the other hand, after a while, the IL-1 chronic production will reprogram the senescence tumors to re-enter the cell cycle, in a condition known as “post-senescence” acquiring stemness properties and promoting tumor relapse (Gelfo et al., 2016; 2018; 2020; Milanovic et al., 2017; Lee and Schmitt, 2019; Saleh et al., 2019; Romaniello et al., 2020; Jochems et al., 2021).

These findings challenge the consolidated theory about senescence as the conclusion of the life cycle followed by permanent growth arrest, and unveil a dynamic post-senescence process, which represents a defy for therapeutical agents. Thus, the contemporary targeting of senescence by senotherapeutic drugs along with target therapy toward ERBB family members may boost the efficacy of future cancer treatment (Zhu et al., 2015; Schafer et al., 2017; Ovadya and Krizhanovsky, 2018; Romaniello et al., 2022).

## References

[B1] Acosta J. C. Banito A. Wuestefeld T. Georgilis A. Janich P. Morton J. P. (2013). A complex secretory program orchestrated by the inflammasome controls paracrine senescence. Nat. Cell Biol. 15, 978–990. 10.1038/ncb2784 23770676 PMC3732483

[B2] Aiello A. Farzaneh F. Candore G. Caruso C. Davinelli S. Gambino C. M. (2019). Immunosenescence and its hallmarks: How to oppose aging strategically? A review of potential options for therapeutic intervention. Front. Immunol. 10, 2247. 10.3389/fimmu.2019.02247 31608061 PMC6773825

[B3] Ansieau S. Bastid J. Doreau A. Morel A. P. Bouchet B. P. Thomas C. (2008). Induction of EMT by twist proteins as a collateral effect of tumor-promoting inactivation of premature senescence. Cancer Cell 14, 79–89. 10.1016/j.ccr.2008.06.005 18598946

[B4] Baselga J. Pfister D. Cooper M. R. Cohen R. Burtness B. Bos M. (2000). Phase I studies of anti-epidermal growth factor receptor chimeric antibody C225 alone and in combination with cisplatin. J. Clin. Oncol. 18, 904–914. 10.1200/jco.2000.18.4.904 10673534

[B5] Beauséjour C. M. Krtolica A. Galimi F. Narita M. Lowe S. W. Yaswen P. (2003). Reversal of human cellular senescence: Roles of the p53 and p16 pathways. EMBO J. 22, 4212–4222. 10.1093/emboj/cdg417 12912919 PMC175806

[B6] Becklund B. R. Purton J. F. Ramsey C. Favre S. Vogt T. K. Martin C. E. (2016). The aged lymphoid tissue environment fails to support naive T cell homeostasis. Sci. Rep. 6, 30842. 10.1038/srep30842 27480406 PMC4969611

[B7] Ben-Porath I. Weinberg R. A. (2005). The signals and pathways activating cellular senescence. Int. J. Biochem. Cell Biol. 37, 961–976. 10.1016/j.biocel.2004.10.013 15743671

[B8] Besancenot R. Chaligné R. Tonetti C. Pasquier F. Marty C. Lécluse Y. (2010). A senescence-like cell-cycle arrest occurs during megakaryocytic maturation: Implications for physiological and pathological megakaryocytic proliferation. PLoS Biol. 8, e1000476. 10.1371/journal.pbio.1000476 20838657 PMC2935456

[B9] Bonilla C. Y. Melo J. A. Toczyski D. P. (2008). Colocalization of sensors is sufficient to activate the DNA damage checkpoint in the absence of damage. Mol. Cell 30, 267–276. 10.1016/j.molcel.2008.03.023 18471973 PMC2879338

[B10] Brennan C. W. Verhaak R. G. W. McKenna A. Campos B. Noushmehr H. Salama S. R. (2013). The somatic genomic landscape of glioblastoma. Cell 155, 462–477. 10.1016/J.CELL.2013.09.034 24120142 PMC3910500

[B11] Carmi Y. Dotan S. Rider P. Kaplanov I. White M. R. Baron R. (2013). The role of IL-1β in the early tumor cell–induced angiogenic response. J. Immunol. 190, 3500–3509. 10.4049/jimmunol.1202769 23475218

[B12] Chakradeo S. Elmore W. Gewirtz A. D. (2015). Is senescence reversible? Curr. Drug Targets 17, 460–466. 10.2174/1389450116666150825113500 26302802

[B13] Chaudhary S. Pothuraju R. Rachagani S. Siddiqui J. A. Atri P. Mallya K. (2021). Dual blockade of EGFR and CDK4/6 delays head and neck squamous cell carcinoma progression by inducing metabolic rewiring. Cancer Lett. 510, 79–92. 10.1016/j.canlet.2021.04.004 33878394 PMC8153085

[B14] Chen J. Zeng F. Forrester S. J. Eguchi S. Zhang M. Z. Harris R. C. (2016). Expression and function of the epidermal growth factor receptor in physiology and disease. Physiol. Rev. 96, 1025–1069. 10.1152/physrev.00030.2015 33003261

[B15] Chou P. Effros B. R. (2013). T cell replicative senescence in human aging. Curr. Pharm. Des. 19, 1680–1698. 10.2174/138161213805219711 23061726 PMC3749774

[B16] Coppé J.-P. Desprez P.-Y. Krtolica A. Campisi J. (2010). The senescence-associated secretory phenotype: The dark side of tumor suppression. Annu. Rev. Pathology Mech. Dis. 5, 99–118. 10.1146/annurev-pathol-121808-102144 PMC4166495 20078217

[B17] Coppé J.-P. P. Patil C. K. Rodier F. Sun Y. Y. Muñoz D. P. Goldstein J. (2008). Senescence-associated secretory phenotypes reveal cell-nonautonomous functions of oncogenic RAS and the p53 tumor suppressor. PLoS Biol. 6, 2853–2868. 10.1371/journal.pbio.0060301 19053174 PMC2592359

[B18] D’Adda Di Fagagna F. (2008). Living on a break: Cellular senescence as a DNA-damage response. Nat. Rev. Cancer 8, 512–522. 10.1038/nrc2440 18574463

[B19] D’Adda Di Fagagna F. Reaper P. M. Clay-Farrace L. Fiegler H. Carr P. Von Zglinicki T. (2003). A DNA damage checkpoint response in telomere-initiated senescence. Nature 426, 194–198. 10.1038/nature02118 14608368

[B20] Di Micco R. Fumagalli M. Cicalese A. Piccinin S. Gasparini P. Luise C. (2006). Oncogene-induced senescence is a DNA damage response triggered by DNA hyper-replication. Nature 444, 638–642. 10.1038/nature05327 17136094

[B21] Di Micco R. Fumagalli M. d’Adda di Fagagna F. (2007). Breaking news: High-speed race ends in arrest--how oncogenes induce senescence. Trends Cell Biol. 17, 529–536. 10.1016/j.tcb.2007.07.012 17980599

[B113] Di Micco R. Krizhanovsky V. Baker D. d’Adda di Fagagna F. (2021). Cellular senescence in ageing: From mechanisms to therapeutic opportunities. Nat. Rev. Mol. Cell Biol. 22, 75–95. 10.1038/s41580-020-00314-w 33328614 PMC8344376

[B22] Dimri G. P. Lee X. Basile G. Acosta M. Scott G. Roskelley C. (1995). A biomarker that identifies senescent human cells in culture and in aging skin *in vivo* . Proc. Natl. Acad. Sci. 92, 9363–9367. 10.1073/pnas.92.20.9363 7568133 PMC40985

[B23] Dulić V. Beney G.-E. Frebourg G. Drullinger L. F. Stein G. H. (2000). Uncoupling between phenotypic senescence and cell cycle arrest in aging p21-deficient fibroblasts. Mol. Cell Biol. 20, 6741–6754. 10.1128/mcb.20.18.6741-6754.2000 10958672 PMC86196

[B24] Elias R. Morales J. Rehman Y. Khurshid H. (2016). Immune checkpoint inhibitors in older adults. Curr. Oncol. Rep. 18, 47. 10.1007/s11912-016-0534-9 27287329

[B25] Espinosa-Cotton M. Rodman S. N. Ross K. A. Jensen I. J. Sangodeyi-Miller K. McLaren A. J. (2019). Interleukin-1 alpha increases anti-tumor efficacy of cetuximab in head and neck squamous cell carcinoma. J. Immunother. Cancer 7, 79. 10.1186/s40425-019-0550-z 30890189 PMC6425573

[B26] Ewald J. A. Desotelle J. A. Wilding G. Jarrard D. F. (2010). Therapy-induced senescence in cancer. J. Natl. Cancer Inst. 102, 1536–1546. 10.1093/jnci/djq364 20858887 PMC2957429

[B27] Ferrara R. Mezquita L. Auclin E. Chaput N. Besse B. (2017). Immunosenescence and immunecheckpoint inhibitors in non-small cell lung cancer patients: Does age really matter? Cancer Treat. Rev. 60, 60–68. 10.1016/j.ctrv.2017.08.003 28889085

[B28] Fisher C. J. Egan M. K. Smith P. Wicks K. Millis R. R. Fentiman I. S. (1997). Histopathology of breast cancer in relation to age. Br. J. Cancer 75, 593–596. 10.1038/bjc.1997.103 9052416 PMC2063298

[B29] Flavell R. A. Sanjabi S. Wrzesinski S. H. Licona-Limón P. (2010). The polarization of immune cells in the tumour environment by TGFbeta. Nat. Rev. Immunol. 10, 554–567. 10.1038/nri2808 20616810 PMC3885992

[B30] Franceschi C. Valensin S. Fagnoni F. Barbi C. Bonafè M. (1999). Biomarkers of immunosenescence within an evolutionary perspective: The challenge of heterogeneity and the role of antigenic load. Exp. Gerontol. 34, 911–921. 10.1016/S0531-5565(99)00068-6 10673145

[B31] Gelfo V. Mazzeschi M. Grilli G. Lindzen M. Santi S. D’Uva G. (2018). A novel role for the interleukin-1 receptor Axis in resistance to anti-EGFR therapy. Cancers (Basel) 1, 1. 10.3390/cancers10100355 PMC6210663 30261609

[B32] Gelfo V. Rodia M. T. Pucci M. Dall’Ora M. Santi S. Solmi R. (2016). A module of inflammatory cytokines defines resistance of colorectal cancer to EGFR inhibitors. Oncotarget 7, 72167–72183. 10.18632/oncotarget.12354 27708224 PMC5342152

[B33] Gelfo V. Romaniello D. Mazzeschi M. Sgarzi M. Grilli G. Morselli A. (2020). Roles of il-1 in cancer: From tumor progression to resistance to targeted therapies. Int. J. Mol. Sci. 21, 6009–6014. 10.3390/ijms21176009 32825489 PMC7503335

[B34] Gibaja A. Aburto M. R. Pulido S. Collado M. Hurle J. M. Varela-Nieto I. (2019). TGFβ2-induced senescence during early inner ear development. Sci. Rep. 9, 5912. 10.1038/s41598-019-42040-0 30976015 PMC6459823

[B35] González-Gualda E. Baker A. G. Fruk L. Muñoz-Espín D. (2021). A guide to assessing cellular senescence *in vitro* and *in vivo* . FEBS J. 288, 56–80. 10.1111/febs.15570 32961620

[B36] Gorgoulis V. Adams P. D. Alimonti A. Bennett D. C. Bischof O. Bishop C. (2019). Cellular senescence: Defining a path forward, Cell. 179. 1. 10.1016/j.cell.2019.10.005 31675495

[B37] Hanna N. Johnson D. Temin S. Baker S. Brahmer J. Ellis P. M. (2017). Systemic therapy for stage IV non–small-cell lung cancer: American society of clinical oncology clinical practice guideline update. J. Clin. Oncol. 35, 3484–3515. 10.1200/JCO.2017.74.6065 28806116

[B38] Hao X. Zhao B. Zhou W. Liu H. Fukumoto T. Gabrilovich D. (2021). Sensitization of ovarian tumor to immune checkpoint blockade by boosting senescence-associated secretory phenotype. iScience 24, 102016. 10.1016/j.isci.2020.102016 33490922 PMC7811168

[B39] Harley C. B. Futcher A. B. Greider C. W. (1990). Telomeres shorten during ageing of human fibroblasts. Nature 345, 458–460. 10.1038/345458a0 2342578

[B40] Hayflick L. Moorhead P. S. (1961). The serial cultivation of human diploid cell strains. Exp. Cell Res. 25, 585–621. 10.1016/0014-4827(61)90192-6 13905658

[B41] Hernandez-Segura A. de Jong T. v. Melov S. Guryev V. Campisi J. Demaria M. (2017). Unmasking transcriptional heterogeneity in senescent cells. Curr. Biol. 27, 2652–2660. 10.1016/j.cub.2017.07.033 28844647 PMC5788810

[B42] Hernandez-Segura A. Nehme J. Demaria M. (2018). Hallmarks of cellular senescence. Trends Cell Biol. 28, 436–453. 10.1016/j.tcb.2018.02.001 29477613

[B43] Holliday R. Tarrant G. M. (1972). Altered enzymes in ageing human fibroblasts. Nature 238, 26–30. 10.1038/238026a0 12635262

[B44] Hotta K. Tabata M. Kiura K. Kozuki T. Hisamoto A. Katayama H. (2007). Gefitinib induces premature senescence in non-small cell lung cancer cells with or without EGFR gene mutation. Oncol. Rep. 17, 313–317. 10.3892/or.17.2.313 17203166

[B45] Huang C. Rajfur Z. Borchers C. Schaller M. D. Jacobson K. (2003). JNK phosphorylates paxillin and regulates cell migration. Nature 424, 219–223. 10.1038/nature01745 12853963

[B46] Huggins C. J. Malik R. Lee S. Salotti J. Thomas S. Martin N. (2013). C/EBPγ suppresses senescence and inflammatory gene expression by heterodimerizing with C/EBPβ. Mol. Cell Biol. 33, 3242–3258. 10.1128/mcb.01674-12 23775115 PMC3753923

[B47] Jochems F. Thijssen B. de Conti G. Jansen R. Pogacar Z. Groot K. (2021). The cancer SENESCopedia: A delineation of cancer cell senescence. Cell Rep. 36, 109441. 10.1016/j.celrep.2021.109441 34320349 PMC8333195

[B48] Jorissen R. N. Walker F. Pouliot N. Garrett T. P. J. Ward C. W. Burgess A. W. (2003). Epidermal growth factor receptor: Mechanisms of activation and signalling. Exp. Cell Res. 284, 31–53. 10.1016/S0014-4827(02)00098-8 12648464

[B49] Justice J. N. Nambiar A. M. Tchkonia T. LeBrasseur N. K. Pascual R. Hashmi S. K. (2019). Senolytics in idiopathic pulmonary fibrosis: Results from a first-in-human, open-label, pilot study. EBioMedicine 40, 554–563. 10.1016/j.ebiom.2018.12.052 30616998 PMC6412088

[B50] Kaplanov I. Carmi Y. Kornetsky R. Shemesh A. Shurin G. v. Shurin M. R. (2019). Blocking IL-1β reverses the immunosuppression in mouse breast cancer and synergizes with anti–PD-1 for tumor abrogation. Proc. Natl. Acad. Sci. 116, 1361–1369. 10.1073/pnas.1812266115 30545915 PMC6347724

[B51] Kovacs E. Zorn J. A. Huang Y. Barros T. Kuriyan J. (2015). A structural perspective on the regulation of the epidermal growth factor receptor. Annu. Rev. Biochem. 84, 739–764. 10.1146/ANNUREV-BIOCHEM-060614-034402 25621509 PMC4452390

[B52] Kowald A. Passos J. F. Kirkwood T. B. L. (2020). On the evolution of cellular senescence. Aging Cell 19, e13270. 10.1111/acel.13270 33166065 PMC7744960

[B53] Krelin Y. Voronov E. Dotan S. Elkabets M. Reich E. Fogel M. (2007). Interleukin-1beta-driven inflammation promotes the development and invasiveness of chemical carcinogen-induced tumors. Cancer Res. 67, 1062–1071. 10.1158/0008-5472.CAN-06-2956 17283139

[B54] Kumari R. Jat P. (2021). Mechanisms of cellular senescence: Cell cycle arrest and senescence associated secretory phenotype. Front. Cell Dev. Biol. 9, 645593. 10.3389/fcell.2021.645593 33855023 PMC8039141

[B55] Laberge R. M. Zhou L. Sarantos M. R. Rodier F. Freund A. de Keizer P. L. J. (2012). Glucocorticoids suppress selected components of the senescence-associated secretory phenotype. Aging Cell 11, 569–578. 10.1111/j.1474-9726.2012.00818.x 22404905 PMC3387333

[B56] Lee J. M. Tsuboi M. Kim E. S. Mok T. S. Garrido P. (2022). Overcoming immunosuppression and pro-tumor inflammation in lung cancer with combined IL-1β and PD-1 inhibition. Future Oncol. 18, 3085–3100. 10.2217/FON-2021-1567 36004638

[B57] Lee S. Schmitt C. A. (2019). The dynamic nature of senescence in cancer. Nat. Cell Biol. 21, 94–101. 10.1038/s41556-018-0249-2 30602768

[B58] Lemmon M. A. Schlessinger J. (2010). Cell signaling by receptor tyrosine kinases. Cell 141, 1117–1134. 10.1016/J.CELL.2010.06.011 20602996 PMC2914105

[B59] Li Y. Zhao H. Huang X. Tang J. Zhang S. Li Y. (2018). Embryonic senescent cells re-enter cell cycle and contribute to tissues after birth. Cell Res. 28, 775–778. 10.1038/s41422-018-0050-6 29872106 PMC6028486

[B60] Lim H. Park H. Kim H. P. (2015). Effects of flavonoids on senescence-associated secretory phenotype formation from bleomycin-induced senescence in BJ fibroblasts. Biochem. Pharmacol. 96, 337–348. 10.1016/j.bcp.2015.06.013 26093063

[B61] Mailand N. Falck J. Lukas C. Syljuåsen R. G. Welcker M. Bartek J. (2000). Rapid destruction of human Cdc25A in response to DNA damage. Science 288, 1425–1429. 10.1126/science.288.5470.1425 10827953

[B62] Maron R. Schechter B. Nataraj N. B. Ghosh S. Romaniello D. Marrocco I. (2019). Inhibition of a pancreatic cancer model by cooperative pairs of clinically approved and experimental antibodies. Biochem. Biophys. Res. Commun. 513, 219–225. 10.1016/J.BBRC.2019.03.204 30952434

[B63] Marrocco I. Romaniello D. Vaknin I. Drago-Garcia D. Oren R. Uribe M. L. (2021). Upfront admixing antibodies and EGFR inhibitors preempts sequential treatments in lung cancer models. EMBO Mol. Med. 13, e13144. 10.15252/EMMM.202013144 33660397 PMC8033519

[B64] Marrocco I. Romaniello D. Yarden Y. (2019). “Cancer immunotherapy: ,” in The dawn of antibody cocktails. 1. 10.1007/978-1-4939-8958-4_2 30539465

[B65] Mastri M. Tracz A. Lee C. R. Dolan M. Attwood K. Christensen J. G. (2018). A transient pseudosenescent secretome promotes tumor growth after antiangiogenic therapy withdrawal. Cell Rep. 25, 3706–3720. 10.1016/j.celrep.2018.12.017 30590043 PMC13277708

[B66] McDermott M. S. J. Conlon N. Browne B. C. Szabo A. Synnott N. C. O’brien N. A. (2019). HER2-targeted tyrosine kinase inhibitors cause therapy-induced-senescence in breast cancer cells. Cancers (Basel) 11, 197. 10.3390/cancers11020197 30743996 PMC6406301

[B67] Milanovic M. Fan D. N. Y. Belenki D. Däbritz J. H. M. Zhao Z. Yu Y. (2017). Senescence-associated reprogramming promotes cancer stemness. Nature 553 (553), 96–100. 10.1038/nature25167 29258294

[B68] Mongiardi M. P. Pellegrini M. Pallini R. Levi A. Falchetti M. L. (2021). Cancer response to therapy-induced senescence: A matter of dose and timing. Cancers (Basel) 13, 484. 10.3390/cancers13030484 33513872 PMC7865402

[B69] Muñoz-Espín D. Cañamero M. Maraver A. Gómez-López G. Contreras J. Murillo-Cuesta S. (2013). Programmed cell senescence during mammalian embryonic development. Cell 155, 1104–1118. 10.1016/j.cell.2013.10.019 24238962

[B70] Noronha A. Belugali Nataraj N. Lee J. S. Zhitomirsky B. Oren Y. Oster S. (2022). AXL and error-prone DNA replication confer drug resistance and offer strategies to treat EGFR-mutant lung cancer. Cancer Discov. 1, OF1–OF18. 10.1158/2159-8290.CD-22-0111 PMC9627128 35895872

[B71] Ovadya Y. Krizhanovsky V. (2018). Strategies targeting cellular senescence. J. Clin. Investigation 128, 1247–1254. 10.1172/JCI95149 PMC5873866 29608140

[B72] Paez-Ribes M. González-Gualda E. Doherty G. J. Muñoz-Espín D. (2019). Targeting senescent cells in translational medicine. EMBO Mol. Med. 11, e10234. 10.15252/emmm.201810234 31746100 PMC6895604

[B73] Palmer S. Albergante L. Blackburn C. C. Newman T. J. (2018). Thymic involution and rising disease incidence with age. Proc. Natl. Acad. Sci. U. S. A. 115, 1883–1888. 10.1073/pnas.1714478115 29432166 PMC5828591

[B74] Park K. Tan E. H. O’Byrne K. Zhang L. Boyer M. Mok T. (2016). Afatinib versus gefitinib as first-line treatment of patients with EGFR mutation-positive non-small-cell lung cancer (LUX-Lung 7): A phase 2B, open-label, randomised controlled trial. Lancet Oncol. 17, 577–589. 10.1016/S1470-2045(16)30033-X 27083334

[B75] Pelissier Vatter F. A. Schapiro D. Chang H. Borowsky A. D. Lee J. K. Parvin B. (2018). High-dimensional phenotyping identifies age-emergent cells in human mammary epithelia. Cell Rep. 23, 1205–1219. 10.1016/j.celrep.2018.03.114 29694896 PMC5946804

[B76] Pili R. Guo Y. Chang J. Nakanishi H. Martin G. R. Passaniti A. (1994). Altered angiogenesis underlying age-dependent changes in tumor growth. J. Natl. Cancer Inst. 86, 1303–1314. 10.1093/jnci/86.17.1303 7520508

[B77] Reyes de Mochel N. Cheong K. N. Cassandras M. Wang C. Krasilnikov M. Matatia P. (2020). Sentinel p16-INK4a+ cells in the basement membrane form a reparative niche in the lung. bioRxiv 1, 1. 10.1101/2020.06.10.142893 PMC10621323 36227993

[B78] Ridker P. M. MacFadyen J. G. Thuren T. Everett B. M. Libby P. Glynn R. J. (2017). Effect of interleukin-1β inhibition with canakinumab on incident lung cancer in patients with atherosclerosis: Exploratory results from a randomised, double-blind, placebo-controlled trial. Lancet 390, 1833–1842. 10.1016/S0140-6736(17)32247-X 28855077

[B79] Romaniello D. Gelfo V. Pagano F. Ferlizza E. Sgarzi M. Mazzeschi M. (2022). Senescence-associated reprogramming induced by interleukin-1 impairs response to EGFR neutralization. Cell Mol. Biol. Lett. 27, 20. 10.1186/s11658-022-00319-7 35236282 PMC8903543

[B80] Romaniello D. Marrocco I. Nataraj N. B. Ferrer I. Drago-Garcia D. Vaknin I. (2020). Targeting her3, a catalytically defective receptor tyrosine kinase, prevents resistance of lung cancer to a third-generation egfr kinase inhibitor. Cancers (Basel) 12, 2394. 10.3390/cancers12092394 32847130 PMC7563838

[B81] Rosemblit C. Datta J. Lowenfeld L. Xu S. Basu A. Kodumudi K. (2018). Oncodriver inhibition and CD4 + Th1 cytokines cooperate through Stat1 activation to induce tumor senescence and apoptosis in HER2+ and triple negative breast cancer: Implications for combining immune and targeted therapies. Available at: www.oncotarget.com. 10.18632/oncotarget.25208 PMC5955413 29796172

[B82] Ruhland M. K. Alspach E. (2021). Senescence and immunoregulation in the tumor microenvironment. Front. Cell Dev. Biol. 9, 754069. 10.3389/fcell.2021.754069 34692707 PMC8529213

[B83] Ruscetti M. Morris J. P. Mezzadra R. Russell J. Leibold J. Romesser P. B. (2020). Senescence-induced vascular remodeling creates therapeutic vulnerabilities in pancreas cancer. Cell 181, 424–441. 10.1016/j.cell.2020.03.008 32234521 PMC7278897

[B84] Russo M. Crisafulli G. Sogari A. Reilly N. M. Arena S. Lamba S. (2019). Adaptive mutability of colorectal cancers in response to targeted therapies. Science 366, 1473–1480. 10.1126/science.aav4474 31699882

[B85] Saleh T. Bloukh S. Carpenter V. J. Alwohoush E. Bakeer J. Darwish S. (2020). Therapy-induced senescence: An “old” friend becomes the enemy. Cancers (Basel) 12, 822. 10.3390/cancers12040822 32235364 PMC7226427

[B86] Saleh T. Tyutynuk-Massey L. Cudjoe E. K. Idowu M. O. Landry J. W. Gewirtz D. A. (2018). Non-cell autonomous effects of the senescence-associated secretory phenotype in cancer therapy. Front. Oncol. 8, 164. 10.3389/fonc.2018.00164 29868482 PMC5968105

[B87] Saleh T. Tyutyunyk-Massey L. Murray G. F. Alotaibi M. R. Kawale A. S. Elsayed Z. (2019). Tumor cell escape from therapy-induced senescence. Biochem. Pharmacol. 162, 202–212. 10.1016/j.bcp.2018.12.013 30576620

[B88] Schafer M. J. White T. A. Iijima K. Haak A. J. Ligresti G. Atkinson E. J. (2017). Cellular senescence mediates fibrotic pulmonary disease. Nat. Commun. 8, 14532. 10.1038/ncomms14532 28230051 PMC5331226

[B89] Shiloh Y. (2006). The ATM-mediated DNA-damage response: Taking shape. Trends Biochem. Sci. 31, 402–410. 10.1016/j.tibs.2006.05.004 16774833

[B90] Singh S. Xiao Z. Bavisi K. Roszik J. Melendez B. D. Wang Z. (2021). IL-1α mediates innate and acquired resistance to immunotherapy in melanoma. J. Immunol. 206, 1966–1975. 10.4049/jimmunol.2000523 33722878 PMC8023145

[B91] Soria J.-C. Ohe Y. Vansteenkiste J. Reungwetwattana T. Chewaskulyong B. Lee K. H. (2018). Osimertinib in untreated EGFR-mutated advanced non-small-cell lung cancer. N. Engl. J. Med. 378, 113–125. 10.1056/NEJMOA1713137 29151359

[B92] Srivastava S. Nataraj N. B. Sekar A. Ghosh S. Bornstein C. Drago-Garcia D. (2019). ETS proteins bind with glucocorticoid receptors: Relevance for treatment of ewing sarcoma. Cell Rep. 29, 104–117. e4. 10.1016/J.CELREP.2019.08.088 31577941 PMC6899513

[B93] Stanam A. Gibson-Corley K. N. Love-Homan L. Ihejirika N. Simons A. L. Stanam A. (2016). Interleukin-1 blockade overcomes erlotinib resistance in head and neck squamous cell carcinoma. Oncotarget 7, 76087–76100. 10.18632/oncotarget.12590 27738319 PMC5342798

[B94] Sugita S. Ito K. Yamashiro Y. Moriya S. Che X. F. Yokoyama T. (2015). EGFR-independent autophagy induction with gefitinib and enhancement of its cytotoxic effect by targeting autophagy with clarithromycin in non-small cell lung cancer cells. Biochem. Biophys. Res. Commun. 461, 28–34. 10.1016/j.bbrc.2015.03.162 25858318

[B95] Thomas R. Wang W. Su D. M. (2020). Contributions of age-related thymic involution to immunosenescence and inflammaging. Immun. Ageing 17, 2. 10.1186/s12979-020-0173-8 31988649 PMC6971920

[B96] Tran P. T. Shroff E. H. Burns T. F. Thiyagarajan S. Das S. T. Zabuawala T. (2012). Twist1 suppresses senescence programs and thereby accelerates and maintains mutant Kras-induced lung tumorigenesis. PLoS Genet. 8, e1002650. 10.1371/journal.pgen.1002650 22654667 PMC3360067

[B97] Turenne G. A. Paul P. Laflair L. Price B. D. (2001). Activation of p53 transcriptional activity requires ATM’s kinase domain and multiple N-terminal serine residues of p53. Oncogene 20, 5100–5110. 10.1038/sj.onc.1204665 11526498

[B98] Vernot J. P. (2020). Senescence-associated pro-inflammatory cytokines and tumor cell plasticity. Front. Mol. Biosci. 7, 63. 10.3389/fmolb.2020.00063 32478091 PMC7237636

[B99] Visconti R. Della Monica R. Grieco D. (2016). Cell cycle checkpoint in cancer: A therapeutically targetable double-edged sword. J. Exp. Clin. Cancer Res. 35, 153. 10.1186/s13046-016-0433-9 27670139 PMC5037895

[B100] Voronov E. Apte R. N. (2020). Targeting the tumor microenvironment by intervention in interleukin-1 Biology. Curr. Pharm. Des. 23, 4893–4905. 10.2174/1381612823666170613080919 28606052

[B101] Wang L. Leite de Oliveira R. Wang C. Fernandes Neto J. M. Mainardi S. Evers B. (2017). High-throughput functional genetic and compound screens identify targets for senescence induction in cancer. Cell Rep. 21, 773–783. 10.1016/j.celrep.2017.09.085 29045843

[B102] Wang M. Morsbach F. Sander D. Gheorghiu L. Nanda A. Benes C. (2011). EGF receptor inhibition radiosensitizes NSCLC cells by inducing senescence in cells sustaining DNA double-strand breaks. Cancer Res. 71, 6261–6269. 10.1158/0008-5472.CAN-11-0213 21852385 PMC3185115

[B103] Wee P. Wang Z. (2017). Epidermal growth factor receptor cell proliferation signaling pathways. Cancers (Basel) 9, 52. 10.3390/CANCERS9050052 28513565 PMC5447962

[B104] Yarden Y. Sliwkowski M. X. (2001). Untangling the ErbB signalling network. Nat. Rev. Mol. Cell Biol. 2, 127–137. 10.1038/35052073 11252954

[B105] Yoneda K. Imanishi N. Ichiki Y. Tanaka F. (2019). Treatment of non-small cell lung cancer with EGFR-mutations. J. UOEH 41, 153–163. 10.7888/JUOEH.41.153 31292359

[B106] Yosef R. Pilpel N. Tokarsky-Amiel R. Biran A. Ovadya Y. Cohen S. (2016). Directed elimination of senescent cells by inhibition of BCL-W and BCL-XL. Nat. Commun. 7, 11190. 10.1038/ncomms11190 27048913 PMC4823827

[B107] Yu Y. Schleich K. Yue B. Ji S. Lohneis P. Kemper K. (2018). Targeting the senescence-overriding cooperative activity of structurally unrelated H3K9 demethylases in melanoma. Cancer Cell 33, 785–336. e8. 10.1016/j.ccell.2018.03.009 29634951

[B108] Zhang B. Fu D. Xu Q. Cong X. Wu C. Zhong X. (2018). The senescence-associated secretory phenotype is potentiated by feedforward regulatory mechanisms involving Zscan4 and TAK1. Nat. Commun. 9, 1723. 10.1038/s41467-018-04010-4 29712904 PMC5928226

[B109] Zhou J. Down J. M. George C. N. Murphy J. Lefley D. v. Tulotta C. (2022). Novel methods of targeting IL-1 signalling for the treatment of breast cancer bone metastasis. Cancers (Basel) 14, 4816. 10.3390/CANCERS14194816 36230739 PMC9561984

[B110] Zhu Y. Tchkonia T. Pirtskhalava T. Gower A. C. Ding H. Giorgadze N. (2015). The achilles’ heel of senescent cells: From transcriptome to senolytic drugs. Aging Cell 14, 644–658. 10.1111/acel.12344 25754370 PMC4531078

[B111] Zitvogel L. Kepp O. Galluzzi L. Kroemer G. (2012). Inflammasomes in carcinogenesis and anticancer immune responses. Nat. Immunol. 13, 343–351. 10.1038/ni.2224 22430787

[B112] Zou L. (2007). Single- and double-stranded DNA: Building a trigger of ATR-mediated DNA damage response. Genes Dev. 21, 879–885. 10.1101/gad.1550307 17437994

